# Polylactide (PLA) Composites Reinforced with Natural Fibrous Filler Recovered from the Biomass of Sorghum Leaves or Stems

**DOI:** 10.3390/ma18194634

**Published:** 2025-10-08

**Authors:** Ryszard Gąsiorowski, Danuta Matykiewicz, Dominika Janiszewska-Latterini

**Affiliations:** 1 Faculty of Mechanical Engineering, Poznan University of Technology, Piotrowo 3, 61-138 Poznań, Poland; 2 Łukasiewicz Research Network—Poznań Institute of Technology, 6 Ewarysta Estkowskiego St., 61-755 Poznań, Poland; dominika.janiszewska@pit.lukasiewicz.gov.pl

**Keywords:** composites, sorghum, PLA, polylactide, mechanical and thermal properties

## Abstract

In response to environmental pressures and the growing demand for sustainable materials, this study investigates the use of lignocellulosic fillers derived from sorghum (*Sorghum bicolor* L. Moench) biomass, specifically stems and leaves, as reinforcements in biodegradable polylactic acid (PLA) composites. The aim was to assess the effect of filler type and content (5, 10, and 15 wt.%) on the physicochemical properties of the composites. Sorghum was manually harvested in Greater Poland, separated, dried, milled, and fractionated to particles <0.25 mm. Composites were produced via extrusion and injection molding, followed by characterization using differential scanning calorimetry (DSC), dynamic mechanical thermal analysis (DMTA), thermogravimetric analysis (TGA), tensile and impact testing, density measurements, optical microscopy, and scanning electron microscopy (SEM). Results showed that stem-based fillers provided a better balance between stiffness and ductility, along with improved dispersion and interfacial adhesion. In contrast, leaf-based fillers led to higher stiffness but greater brittleness and agglomeration. All composites exhibited decreased impact strength and thermal stability compared to neat PLA, with the extent of these decreases depending on the filler type and loading. The study highlights the potential of sorghum stems as a viable, renewable reinforcement in biopolymer composites, aligning with circular economy and bioeconomy strategies.

## 1. Introduction

The increasing environmental and economic pressures of the 21st century have intensified the global transition from linear production models to a circular economy (CE). A fundamental principle of the CE is the cascading use of materials, where waste is not simply discarded but treated as a secondary raw material for further processing before being recovered as energy. Nevertheless, in Poland, combustion remains the most common method of managing agricultural biomass waste, particularly from cereals and forage crops. This underscores the urgent need to develop effective material recovery strategies and to identify alternative uses for plant residues, especially those that remain unutilized after harvest.

At the same time, growing concerns regarding plastic pollution have accelerated the search for biodegradable alternatives to conventional thermoplastics. Nearly 400 million tons of plastic are produced annually worldwide, yet only about 9% is effectively recycled. Currently, bioplastics represent less than 1% of the total plastics market. Forecasts, however, predict a growth in market value from USD 11.2 billion in 2021 to over USD 46 billion by 2030, driven by regulatory pressure and corporate sustainability commitments [1,2].

A particularly promising class of materials is natural fibre-reinforced composites, which combine polymer matrices with renewable lignocellulosic fillers. These composites offer improved environmental profiles and, in many cases, enhanced mechanical performance. One of the sources of lignocellulose is bagasse, the fibrous residue from sugarcane juice extraction, which is widely used in packaging as pulp for molding. However, due to climatic limitations, sugarcane cultivation is not economically viable in Poland. As a result, interest has been directed towards sorghum (*Sorghum bicolor* L. Moench), a resilient cereal crop well-adapted to Poland’s agro-climatic conditions, which is increasingly recognized as a local and sustainable biomass alternative. Although currently cultivated on a relatively small scale (~100 ha), sorghum is gaining interest due to its high drought resistance, low input requirements, and high biomass yield, particularly under changing climatic conditions [3,4].

Importantly, sorghum biomass, including its leaves and stems, has a high content of cellulose, hemicellulose, and lignin, comparable to, or even exceeding, maize [5]. This biochemical profile makes sorghum a strong candidate for conversion into natural fibrous fillers for polymer composites, with potential applications in packaging, construction, automotive components, and consumer goods. Moreover, unlike maize, where cobs form along the plant’s height, requiring the entire stem to be harvested, sorghum produces compact grain panicles at the tops of the plant, allowing for selective grain harvesting with minimal disturbance to the vegetative biomass, which can subsequently be collected, dried, and mechanically processed [5,6].

Leaves and stems of grain sorghum are mostly left to decompose in the field; however, they hold potential for valorization through mechanical pretreatment (e.g., milling, drying, and sieving) to be used as fillers in thermoplastic or thermosetting polymer matrices. According to the literature, the morphological parts of plants differ in their chemical composition, including cellulose, hemicellulose, lignin, starch, and sugar content, which may significantly influence their compatibility with various thermoplastic matrices [7]. Thermoplastic composite matrices are typically composed of either synthetic polymers, such as polyolefins like polypropylene (PP) and polyethylene (PE), or bio-based polymers, including polylactic acid (PLA). While these materials share many common characteristics, differences in specific properties make them suitable for distinct applications. One such distinguishing property is the material’s hydrophobicity [8]. This parameter is particularly significant in the fabrication of composite materials reinforced with natural fillers, as the latter are predominantly hydrophilic in nature. This disparity may lead to partial or complete incompatibility between the matrix and the filler components [9,10,11]. As a consequence, the resulting composites may exhibit unsatisfactory performance, especially in terms of mechanical properties. However, such limitations can be mitigated through various physical and chemical modifications of the lignocellulosic material [12,13,14,15,16,17]. Moreover, properly applied modifications not only improve interfacial compatibility but can also enhance other performance parameters of the composite, such as flammability resistance [18].

Thanks to its seasonal availability, fast growth cycle, and adaptability, sorghum is positioned as a strategic renewable resource within the context of European bioeconomy strategies. With proper processing, its biomass (particularly leaves and stems) can be integrated into composite material production chains, supporting the development of green, functional materials with a reduced carbon footprint [19].

Studies have shown that such natural fibres can improve the mechanical strength, stiffness, and biodegradability of polymeric materials, while also reducing their environmental impact [20]. Furthermore, although the introduction of lignocellulosic fillers may accelerate thermal degradation, it can also lead to increased char residue and reduced flammability, which may be a key advantage in specific applications [21]. In the development of natural fibre-reinforced composites, biodegradable thermoplastics, particularly polylactic acid (PLA), are increasingly being considered as matrix materials [22,23,24]. Although the mechanical properties of PLA classify it as an engineering plastic, its broader applicability is limited by certain drawbacks, such as low thermo-mechanical stability and high brittleness when compared with polyolefin-based alternatives [25].

However, preparing bio-based composites with stable mechanical properties using natural fillers still poses a major challenge. Therefore, this study investigated the feasibility of producing PLA-based polymer composites reinforced with fibrous fillers derived from sorghum biomass, with a particular focus on leaves and stems as readily available, local, and environmentally friendly reinforcing agents. The composites were prepared using sorghum material that had been previously separated into leaves and stems, continuing earlier research that demonstrated differences in the chemical composition of the plant’s morphological parts [26]. The proposed approach of preparing composites separately using sorghum leaves and stems represents an innovative aspect of this study, as sorghum biomass is typically utilized as a filler without distinguishing between the plant’s morphological parts, often used as an unseparated mixture. This separation and comparative analysis of sorghum’s morphological fractions not only provides new insight into how the plant’s internal structural variation affects composite behavior but also fills a gap in the current literature. While sorghum biomass has been sporadically reported as a natural filler, it is almost exclusively treated as a homogenous material. To the best of our knowledge, this is the first study to evaluate PLA composites reinforced separately with sorghum leaves and stems, allowing for a more nuanced understanding of their individual reinforcement potential. This approach offers a novel contribution to the field of bio-based composites by supporting material selectivity, optimizing filler performance, and promoting the valorization of agricultural residues in line with sustainable development and circular economy goals.

## 2. Materials and Methods

### 2.1. Materials

**Figure 1:**
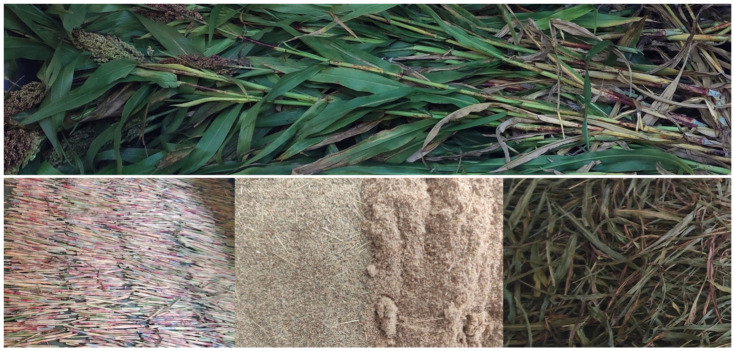
Sorghum after harvest, after separation into stems and leaves, and shredding (<0.25 mm fractions).

Sorghum of the Emeraude variety was used as the research material, sourced from a Polish cultivation located in the Greater Poland region. Manual harvesting was carried out in October 2024, one week before the scheduled mechanical harvest. The plants were cut approximately 15 cm above ground level, without any prior selection, from a designated area indicated by the crop manager. The average plant height was 2.20 m, and the average weight of a single plant was 210 g. A total of approximately 400 plants were harvested, yielding 91 kg of fresh biomass from an area of 20 m^2^, which corresponds to a productivity of 4.55 kg/m^2^ at a material moisture content of 75%. The photo of the sorghum immediately after harvest and after separation into stems (S) and leaves (L), and selected fractions used for producing composites, is shown in Figure 1.

In laboratory conditions, the harvested biomass was manually separated into stems, leaves, and panicles with seeds. The proportions of each component in the total mass were as follows: stems, 47%; leaves, 50%; and seeds, 3%. The material was then dried in a ventilated room maintained at a constant temperature of 22 °C, simulating typical ambient storage conditions. This approach was chosen intentionally in order to avoid additional technological and energy-related costs that might be incurred by a potential manufacturer. It also enabled the assessment of the drying process duration under real-world conditions, as well as its possible impact on material degradation and the quality of the final product. During the drying process, the material was regularly turned, and any parts showing signs of mold growth were removed (this phenomenon occurred only in the stems, particularly in their lower sections). Drying continued until the material reached a moisture content below 15%, suitable for further processing, which took 40 days. The biomass loss due to fungal activity was estimated at approximately 1% of the total mass.

After drying, the separated stems and leaves were subjected to shredding using a Condux impact mill equipped with a cyclone separator. The panicles with seeds were not milled. The material was fractionated using a Fritsch sieving analyzer with mesh sizes of 8 mm, 2 mm, 1 mm, 0.5 mm, and 0.25 mm.

For the material derived from stems, the distribution of particle size fractions (by total mass) was as follows: <0.25 mm—20%, 0.25–0.5 mm—8%, 0.5–1 mm—28%, 1–2 mm—27%, 2–8 mm—16%, and >8 mm—1%. For the material derived from leaves, the fractions were: <0.25 mm—4%, 0.25–0.5 mm—13%, 0.5–1 mm—24%, 1–2 mm—34%, 2–8 mm—25%, and >8 mm—less than 1%. For further research related to the development of composite materials, the fraction containing particles smaller than 0.25 mm was selected.

Polylactic acid (PLA) used in this study was Ingeo™ 2003D (NATURAL grade) supplied by NatureWorks LLC (Blair, Nebraska, USA). It is a commercial-grade, semi-crystalline PLA designed primarily for extrusion and thermoforming applications. According to the manufacturer’s data, the material has a density of 1.24 g/cm^3^, a melt flow index (MFI) of 6 g/10 min (at 210 °C/2.16 kg), and a D-isomer content of approximately 1.4%, providing a balance between processability and mechanical performance.

### 2.2. Sample Preparation

Composite preparation was carried out using a RobotDigg (Shanghai, China) SJ35 laboratory single-screw extruder. The extrusion process was conducted at temperatures ranging from 150 °C to 170 °C, depending on the material composition. The screw rotation speed was set between 5 and 15 rpm, allowing for the effective plasticization of the PLA matrix and the uniform dispersion of the fibrous phase. Composites were prepared with 5%, 10%, and 15% of lignocellulosic filler derived from sorghum leaves (L) or stems (S). After extrusion, the material was cooled with an air stream and subsequently granulated to facilitate further processing. The originally obtained granulate was extruded again to homogenize the material.

Test specimens were produced using a RobotDigg (Shanghai, China) RD-IM240 laboratory injection molding machine. The injection process was conducted within a temperature range of 165–185 °C, with an injection time of 2–4 s, and the injection pressure was set to 0.5 MPa, followed by a holding pressure of 0.6 MPa. The mold temperature was maintained at 35 °C, and the cooling time after injection was 30 s. These parameters were selected based on preliminary trials to ensure proper mold filling and dimensional stability of the samples. Composite pellets based on PLA with 5–15 wt.% lignocellulosic filler derived from sorghum leaves or stems were molded by injection. The injection time and pressure were selected to ensure proper mold filling and minimize surface defects in the specimens. After molding, the samples were cooled inside the mold for a duration sufficient to achieve dimensional stability before ejection. The samples were described according to the filler content leaves (L) or stems (S) as: PLA; PLA5%L; PLA10%L; PLA15%L; PLA5%S; PLA10%S; PLA15%S.

### 2.3. Characterization

The thermal properties of the materials were assessed using differential scanning calorimetry (DSC) with a Phoenix DSC 204 F1 Netzsch (Selb, Germany). The samples of approximately 5.0 ± 0.1 mg were placed in aluminum crucibles with pierced lids, heated up to 180 °C, held in a molten state for 5 min, and cooled to 20 °C at a rate of 10 °C/min and under a nitrogen flow of 25 mL/min. The glass transition temperature was determined from the inflection of the DSC curves. The heating/cooling cycle was repeated twice.

The crystallization degree (X_c_) was calculated according to Equation (1) [27]:

(1) $$Xc=\frac{∆Hm-\Delta Hcc}{∆HmPLA(1-\varphi )}*100$$

where

ΔH_m_ is the melting enthalpy of a sample,

ΔH_cc_ is the cold crystallization enthalpy of a sample,

ΔHmPLA is the melting enthalpy corresponding to a 100% crystalline polylactide (93 J/g) [28].

The thermomechanical characteristics of the composites were assessed using dynamic mechanical thermal analysis (DMTA) in torsion mode (Anton Paar (Graz, Austria) MCR 301) at a frequency of 1 Hz, over a temperature range of 25 to 120 °C, with a heating rate of 2 °C/min. The glass transition temperature (T_g_) was indicated at the maximum value of tan δ.

Density of the materials was determined using AXIS AD200 scales (Gdańsk, Poland), in accordance with the ISO 1183-1 standard [29].

Charpy impact tests were carried out in accordance with ISO 179-1 [30] using a Zwick/Roell (Ulm, Germany) 5109 pendulum impact tester. The tests were performed on unnotched specimens with standard dimensions of 80 × 10 × 5 mm, as specified in ISO 179-1, under controlled laboratory conditions (23 ± 2 °C, 50 ± 5% relative humidity). An impact energy of 5 J was selected based on the material type to ensure complete fracture of the specimens. A minimum of four repetitions was conducted for each material set to provide the statistical reliability of the results.

Tensile tests were performed in accordance with ISO 527-1 [31] using a Zwick (Ulm, Germany) Z010 universal testing machine equipped with an extensometer. Standard type 1A specimens were tested under controlled laboratory conditions (23 ± 2 °C, 50 ± 5% relative humidity). A testing speed of 1 mm/min was applied for the determination of the tensile modulus, followed by 50 mm/min for measuring tensile strength and elongation at break, in accordance with the standard. At least five repetitions were carried out for each material variant to ensure statistical reliability of the results.

Thermogravimetric analysis (TGA) was used to evaluate the thermal stability of the materials. The test was conducted using 10 mg samples in ceramic vessels under nitrogen and air atmospheres, ranging from 30 to 900 °C at a rate of 10 °C/min, with a gas flow of 25 mL/min (Netzsch TG 209 F1 apparatus, Selb, Germany). The temperature at which mass loss was 5 (T5%) and 10% (T10%), residual mass at 900 °C (W%), and maximum thermal degradation temperatures from derived thermogravimetric graphs (DTG) were determined.

Microscopic observations were carried out on the cross-sections of the samples using a Zeiss (Oberkochen, Germany) STEMI 2000C stereomicroscope under reflected light. Images were captured at magnifications appropriate for the examined structures to assess internal morphology and identify possible defects within the material.

The morphology of the fracture surfaces was analyzed using a scanning electron microscope (SEM Inspect S, FEI (Waltham, Massachusetts, USA)). Before observation, the samples were coated with a thin conductive layer of gold and platinum using a sputter coater to improve image quality and prevent charging during analysis. SEM images were collected under high-vacuum conditions at an accelerating voltage suitable for polymer-based materials.

## 3. Results and Discussion

### 3.1. Differential Scanning Calorimetry (DSC)

**Figure 2:**
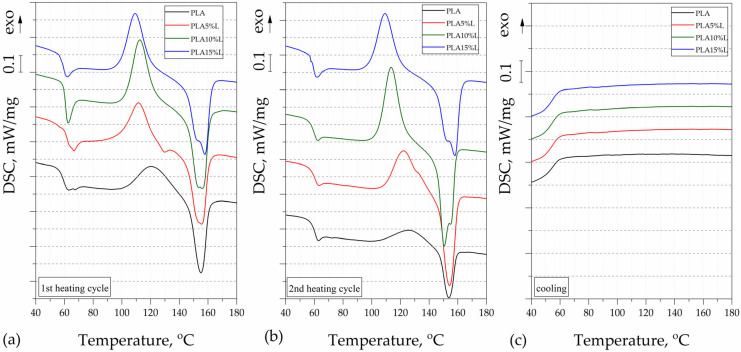
DSC thermograms of PLA and PLA composites with leaves for (**a**) first heating cycle, (**b**) second heating cycle, and (**c**) cooling stage.

**Figure 3:**
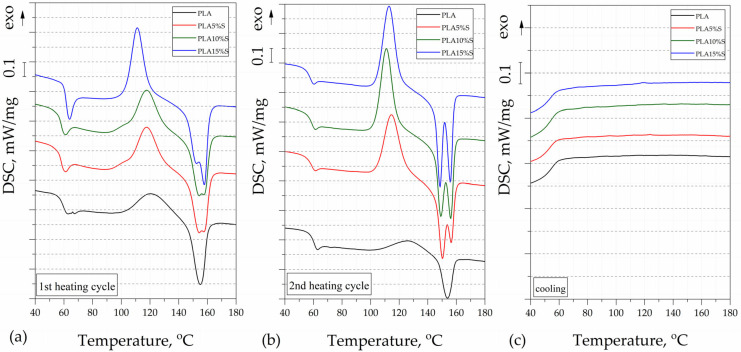
DSC thermograms of PLA and PLA composites with stems for (**a**) first heating cycle, (**b**) second heating cycle, and (**c**) cooling stage.

The thermal properties of the PLA-based composites were evaluated using differential scanning calorimetry, and the results, presented in the form of thermograms from the 1st heating, 2nd heating, and cooling cycles, are shown in Figure 2 and Figure 3. The DSC thermal parameters such as glass transition temperature (T_g_), melting temperature (T_M_), and cold crystallization temperature (T_CC_), determined from the 1st and 2nd heating, are collected in Table 1. A slight decrease in T_g_ was noted for all composites filled with stems. Different effects of the incorporated stem and leaf fibers on the thermal properties, as determined by DSC, can be observed. This phenomenon may result from the different structure and organic compound content of this type of filler, especially waxes occurring on the surface of leaves [11,22].

Moreover, the cold crystallization temperature (T_CC_) for this type of material was lower than for the unmodified PLA matrix. The high amount of stems (10, 15%) and leaves (15%) fibers facilitated the nucleation in the composites, hence decreasing the cold crystallization temperature of the polylactide matrix. The highest Xc indicated from the second heating curves values was indicated for PLA10%S (3.39%), PLA15%S (2.71%), and PLA15%L (1.35%). Data from the second heating cycle were analyzed to exclude the influence of process conditions and to compare the material structure. No significant differences were observed in the cooling curves. The used polylactide is characterized by an almost amorphous structure in the solidified [32].

For PLA10%L and PLA15%L samples, two peaks (T_M1_ and T_M2_) at 155.8 °C/151.8 and at 150.5 °C/158.0 were visible, respectively. Another specific melting behavior was observed for samples with filler in the form of stems. These composites exhibit two sharp melting peaks in the DSC curves. According to the literature, within the complex melting region, the melting of low-quality or metastable crystals may occur, accompanied by partial recrystallization, and ultimately, overall melting [33,34]. Unmodified PLA indicated a low melting enthalpy, which results from the very low rate of crystallization of the specific type of PLA [35]. The composite samples showed very low crystallinity and the materials may be considered as almost amorphous.

### 3.2. Dynamic Mechanical Thermal Analysis (DMTA)

**Figure 4:**
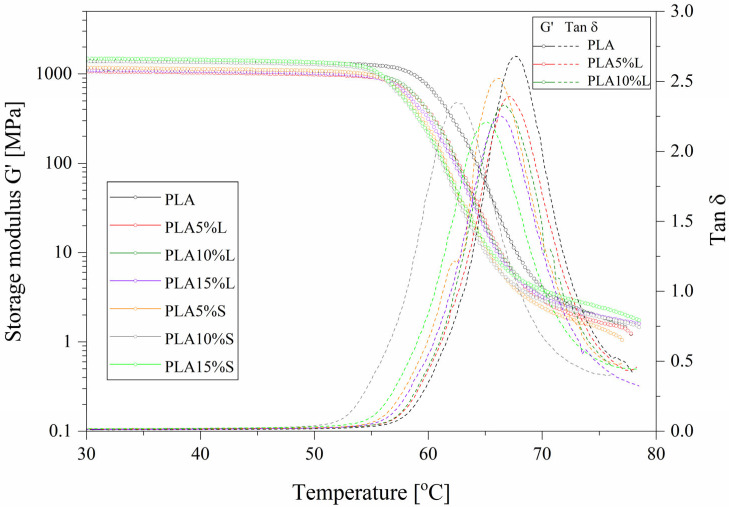
DMTA storage modulus and damping factor vs. temperature plots of PLA and PLA composites.

The influence of the addition of natural filler on the thermomechanical properties of composites with a PLA matrix was evaluated. The assessment of thermomechanical properties under dynamic load conditions (DMTA) is shown in the form of plots of changes in storage modulus (G’) and damping factor (tanδ) vs. temperature in Figure 4. The glass transition temperature (T_g_) was determined as a peak of the tanδ curve, and thermomechanical parameters are collected and presented in Table 2. In the case of composites with natural fillers, changes in the G’ value are observed. For PLA 15%S samples, the highest storage modulus associated with an increase in stiffness can be observed across the entire temperature range considered. The increase was observed together with the increase in the content of stems in the polymer matrix. For all composites filled with stems, slightly lower T_g_ values were noted, which is consistent with the results of DSC analysis. The glass transition temperatures for all the tested materials were comparable and ranged from 66 to 68 °C. This proved that the addition of natural fillers did not significantly affect the T_g_ [36,37].

### 3.3. Mechanical Properties and Density

**Figure 5:**
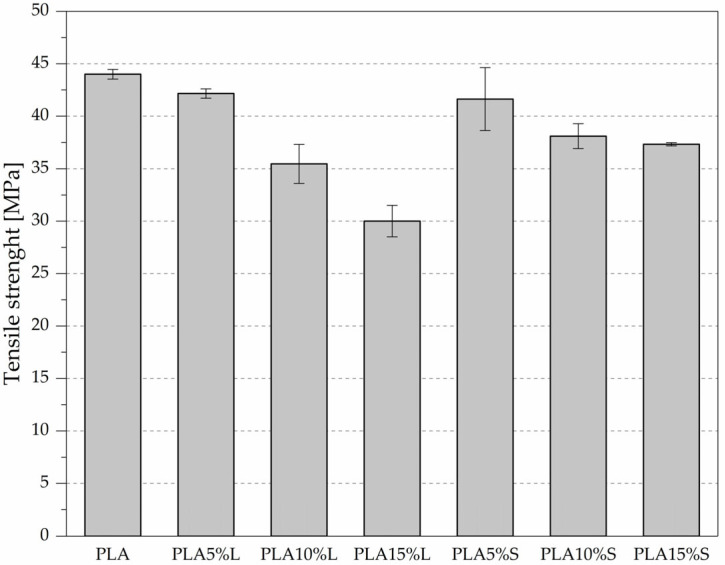
Tensile strength of PLA composites with fibrous filler from sorghum leaves (L) and stems (S)—tensile test results according to ISO 527.

**Figure 6:**
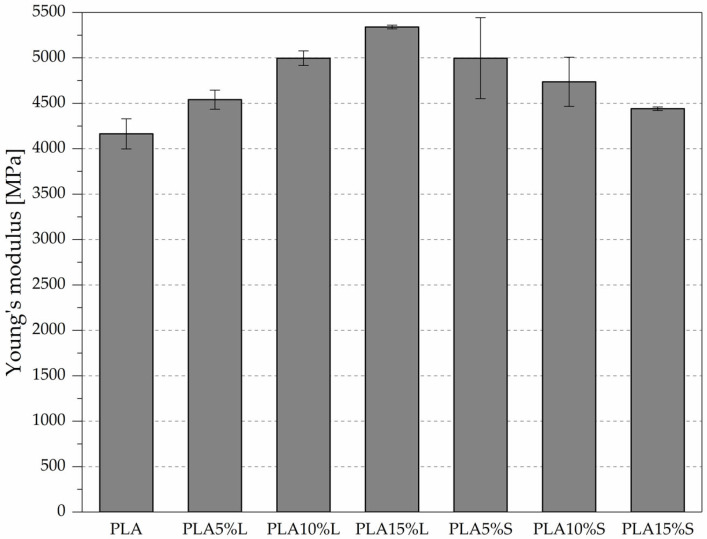
Young’s modulus of PLA composites with fibrous filler from sorghum leaves (L) and stems (S)—results from tensile test according to ISO 527.

The mechanical properties of PLA-based composites reinforced with natural fibrous fillers were evaluated in accordance with ISO 527; the results are presented in Figure 5 and Figure 6 and Table 3.

Pure PLA exhibited a tensile strength of 44.00 MPa, a Young’s modulus of 4163 MPa, and an elongation at break of 2.40%, serving as a reference for assessing the effects of filler incorporation.

The addition of leaf-derived filler (L) led to a progressive increase in Young’s modulus, reaching 5340 MPa at 15% filler content. Notable reduction in tensile strength, from 42.17 MPa (at 5%) to 30.00 MPa (at 15%), was observed. A similar trend was observed in the elongation at break, which decreased from 2.13% to 0.86%, indicating reduced ductility with increasing filler content. PLA reinforced with sorghum leaf filler (L) demonstrated a progressive increase in stiffness, as indicated by a rise in Young’s modulus from 4540 MPa (5% L) to 5340 MPa (15% L), suggesting improved rigidity due to the filler’s reinforcing effect. However, this enhancement came at the expense of tensile strength and ductility. The tensile strength significantly decreased from 42.17 MPa at 5% to 30.00 MPa at 15%, while elongation at break dropped sharply from 2.13% to 0.86%, indicating increased brittleness and reduced strain-bearing capacity. These trends suggest a potential deterioration in interfacial adhesion and filler agglomeration at higher concentrations, which could negatively impact stress transfer.

For the stem-based filler (S), Young’s modulus increased at 5% filler content (4997 MPa) and then gradually decreased at 10% and 15% filler (to 4737 MPa and 4440 MPa, respectively). The tensile strength showed a similar trend, with a moderate decrease from 41.63 MPa (at 5%) to 37.33 MPa (at 15%). The elongation at break remained relatively stable at 1.5% to 1.4%, which is higher than in the leaf-filled composites at concentrations of 10% and 15%. Composites containing stem-derived filler (S) showed a more balanced mechanical response—Young’s modulus was highest at 5% (4997 MPa), and gradually decreased with increasing concentration (4737 MPa at 10% and 4440 MPa at 15%), possibly due to less effective filler dispersion or weaker interfacial bonding. However, tensile strength and strain at break were less adversely affected. The tensile strength remained relatively high (from 41.63 MPa at 5% to 37.33 MPa at 15%), and elongation at break remained above 1.4% at all filler levels, indicating that stem fillers better preserved the composite’s ductility.

Overall, the results suggest that while both fillers can increase the stiffness of PLA, leaf-based fillers induce greater embrittlement and strength loss at higher concentrations, whereas stem-based fillers offer a more favorable balance between stiffness and ductility, making them potentially more suitable for applications requiring moderate mechanical performance without excessive brittleness. The reduction in tensile strength may be caused by inadequate particle dispersion, insufficient wetting of the particles within the matrix, and poor matrix particle adhesion [12].

**Figure 7:**
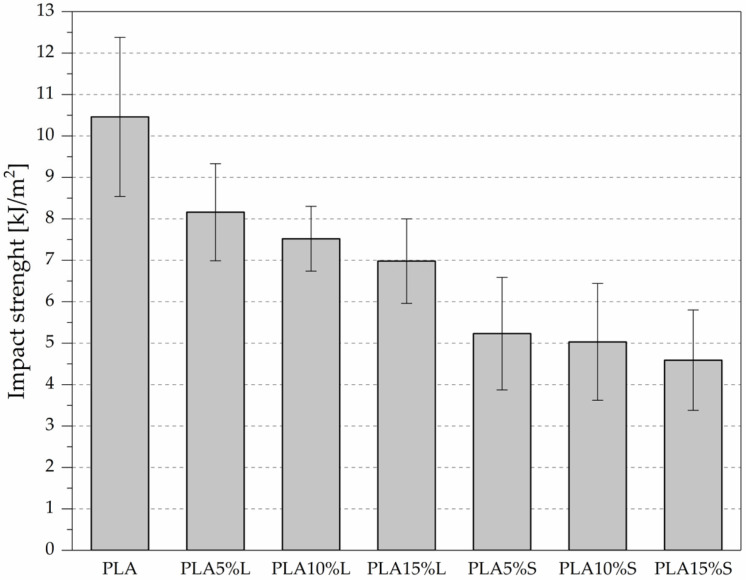
Impact strength of PLA composites with fibrous fillers from sorghum leaves (L) and stems (S).

The impact strength of the PLA-based composites was assessed to evaluate their resistance to dynamic loading; the results are presented in Figure 7 and Table 4.

The unfilled PLA exhibited the highest impact strength, measured at 10.46 ± 1.92 kJ/m^2^, serving as a reference for evaluating the effect of lignocellulosic filler addition. For leaf-derived composites (L), impact strength decreased from 8.16 ± 1.17 kJ/m^2^ at 5% filler loading to 6.98 ± 1.02 kJ/m^2^ at 15%. Although this represents a notable decline, the reduction is less pronounced compared to stem-based composites, indicating a better energy absorption capacity and potentially a more favorable filler-matrix interaction in the case of leaf fibers. Stem-derived composites (PLA-S) showed consistently lower impact resistance across all filler contents, with values dropping from 5.23 ± 1.36 kJ/m^2^ (5%) to 4.59 ± 1.21 kJ/m^2^ (15%). This trend suggests poorer dispersion or adhesion of the stem fibers within the PLA matrix, which may act as stress concentrators and promote crack initiation under impact loading.

The incorporation of sorghum-derived fillers, regardless of their origin, resulted in a progressive reduction in impact strength with increasing filler content. The results confirm that the addition of sorghum fillers leads to a decline in impact toughness, with leaf fillers offering superior performance over stem fillers. The observed reductions in impact strength are typical for natural fiber-reinforced composites and can be attributed to the inherently brittle nature of the PLA matrix, combined with potential microstructural defects introduced during the incorporation of the filler. The reduction in impact strength can also be attributed to the stiffness of the sorghum filler fibres and incomplete wetting of the fibres by the PLA polymer, which results from the hydrophilic nature of natural fillers (greater in the case of stems, because there are hydrophobic waxes on the surface of the leaves) and the hydrophobic nature of PLA. This can result in poor interfacial bonding and strength with increased filler content [11].

**Figure 8:**
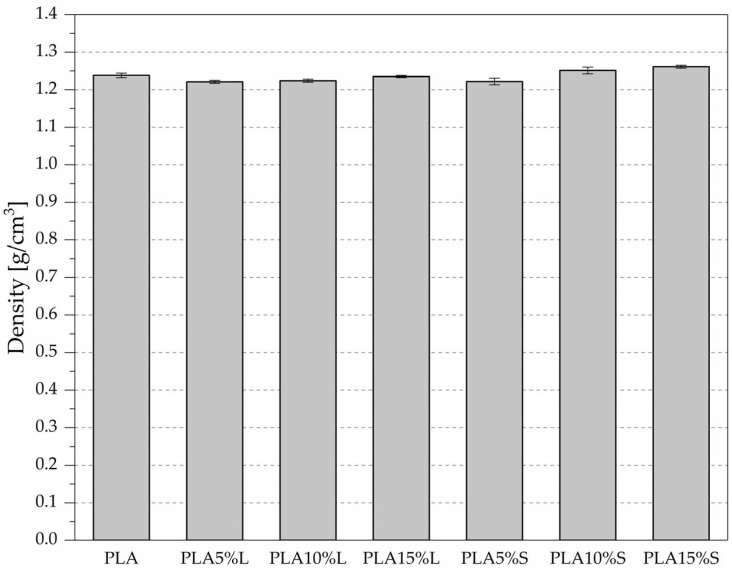
Average density of PLA composites with sorghum leaf (L) and stem (S) fibrous fillers.

The average density of neat PLA was 1.2382 g/cm^3^, serving as a reference point for evaluating the influence of fibrous fillers derived from sorghum leaves (L) and stems (S). The results are presented in Figure 8 and Table 5.

The addition of leaf-based filler in amounts ranging from 5% to 15% resulted in a slight decrease in material density—from 1.2214 g/cm^3^ (5%) to 1.2345 g/cm^3^ (15%). This suggests that the fibrous material from leaves has a lower density than PLA, causing a slight dilution of the polymer matrix. In contrast, composites containing stem-based filler showed a systematic increase in density with rising filler content. The average density of the PLA15%S sample reached 1.2606 g/cm^3^, which is higher than neat PLA. This indicates that the stem-derived filler is either denser than the leaf-derived one or contributes more significantly to the compaction of the composite structure (e.g., through lower porosity or better particle packing).

These results confirmed that the type and content of natural fibrous filler have a significant influence on the composite structure. Composites with leaf-derived filler showed a slight reduction in density compared to neat PLA, which may be attributed to the lower bulk density of the filler and its potential to dilute the polymer matrix. In contrast, stem-derived fillers led to a consistent increase in density with rising content, indicating better structural packing or higher intrinsic density of the filler. These findings suggest that the filler morphology and distribution play a crucial role in shaping the final properties of natural fibre-filled composites and should be carefully considered in material design.

### 3.4. Thermogravimetry

Thermal stability of PLA-based composites containing natural fibrous fillers derived from sorghum leaves (L) and stems (S) was evaluated under nitrogen and air atmospheres; the results are presented in Table 6. Neat PLA exhibited the highest thermal stability, with initial decomposition temperatures (T5%) of 330.7 °C in nitrogen and 322.5 °C in air. The introduction of natural fillers resulted in a clear reduction in thermal stability across all tested variants.

In nitrogen, T5% decreased significantly with increasing filler content. For leaf-derived composites, T5% dropped from 288.4 °C (PLA5%L) to 285.1 °C (PLA10%L), with a slight increase at PLA15%L (290.9 °C), indicating a moderate influence of the filler. In contrast, stem-derived composites showed a more pronounced reduction, with PLA15%S exhibiting the lowest T5% (269.1 °C). Similar trends were observed for T10% and DTG peak temperatures, confirming earlier onset and faster degradation of the filled composites. DTG peak temperatures decrease with increasing filler content, particularly for stem-derived composites, further confirming the earlier onset and more rapid progress of thermal degradation. For PLA15%S, DTG peaks at only 320 °C (N_2_) and 321 °C (air) were observed, compared to 367 °C and 362 °C for neat PLA. Under oxidative (air) conditions, all samples degraded at slightly lower temperatures than under nitrogen conditions. PLA15%S again exhibited the lowest thermal stability (T5% = 270.4 °C).

The introduction of natural fibrous fillers, both from the leaves (L) and stems (S) of sorghum, leads to a noticeable reduction in the onset degradation temperatures (T5% and T10%) in both atmospheres, indicating a decrease in thermal stability. This effect is more pronounced for stem-based fillers, especially at higher contents (e.g., PLA15%S: T5% = 269.1 °C in nitrogen, 270.4 °C in air), suggesting that stem fillers may contain more thermally unstable components such as hemicellulose or lignin residues.

Residual mass at 900 °C was significantly higher for composites than for neat PLA, indicating char formation promoted by the presence of lignocellulosic material. The highest residue in air was noted for PLA15%S (3.52%), suggesting that stem-derived fillers leave more thermally stable inorganic or carbonaceous residues, possibly due to their lignin content.

The obtained thermogravimetric analysis results (DTG Peak [°C]/max. rate [%/min]) indicate that the thermal decomposition of the composites proceeds more slowly compared to the neat polymer. This phenomenon can be attributed to the presence of lignocellulosic substances (such as lignin) in the fillers, which undergo charring during decomposition. The resulting char layer acts as a barrier, limiting oxygen access and heat transfer, thereby slowing down the combustion process [38]. This effect is clearly correlated with the filler content—higher filler concentrations lead to increased char formation and a more pronounced delay in degradation.

**Figure 9:**
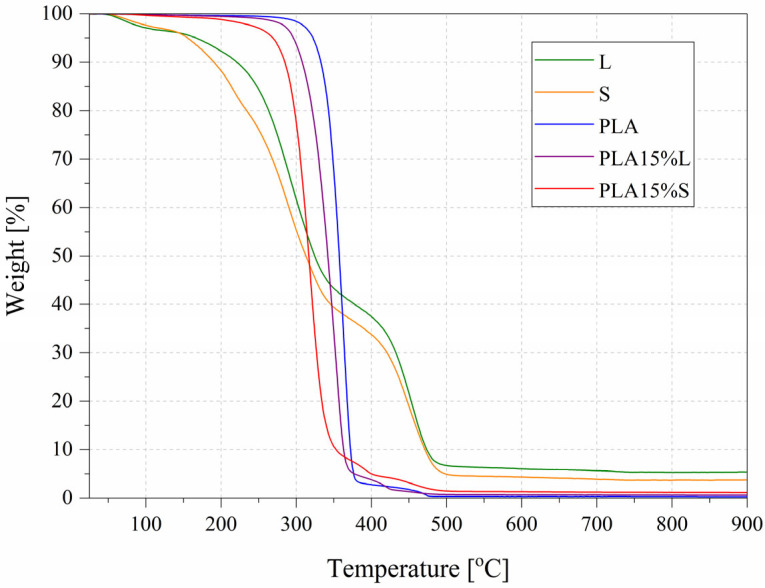
Representative TGA curves in air atmosphere of PLA, fillers and PLA composites.

Representative TGA curves (in air atmosphere) presented in Figure 9 cover the neat PLA, as well as PLA15%L and PLA15%S composites, along with the individual fillers L and S.

As can be observed, the thermal decomposition of the filled composites starts at a lower temperature compared to neat PLA, and the process becomes clearly multi-step, in contrast to the single-stage, intense degradation of pure PLA. These differences are consistent with the expected thermal behavior of hybrid systems containing organic fillers.

Although the fillers (L and S) themselves degrade at much lower temperatures, the composites contain only 15 wt% of these fillers, and the PLA matrix largely governs their thermal behavior. Consequently, the T5% and T10% values for the composites are lower than those of pure PLA, but significantly higher than those of fillers only.

In summary, natural fibrous fillers influence the thermal stability of PLA composites, especially those derived from stems. Leaf-derived fillers exhibit a slightly less pronounced reduction, which may be attributed to their lower thermal reactivity or better dispersion within the matrix. The extent of thermal degradation clearly depends on both the filler type and its content, with leaf-derived fillers showing a milder effect compared to stem-derived ones. This behavior is likely related to the distinct chemical compositions of sorghum leaves and stems. Both types of fillers have comparable cellulose content, but stems tend to contain higher amounts of lignin and hemicellulose [39]—components that degrade at lower temperatures.

### 3.5. Microscopic Analysis

**Figure 10:**
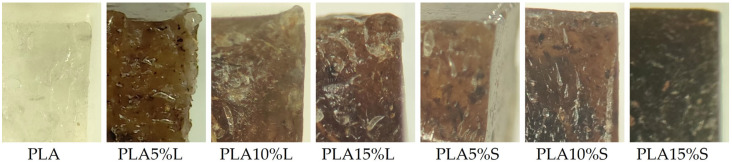
Optical microscope images of fracture surfaces of neat PLA and PLA composites reinforced with 5%, 10%, and 15% sorghum leaf (L) or stem (S) fibrous fillers.

The images provided in Figure 10 show PLA-based composites filled with 5%, 10%, and 15% (by weight) of natural fibrous fillers derived from sorghum leaves (L) and stems (S). The prepared samples measured approximately 1 × 1 × 1 mm, and the images were taken at 2× magnification. For comparison, a reference image of neat PLA is also included.

Microscopic observations of PLA composites reinforced with fibrous fillers derived from sorghum leaves and stems reveal distinct differences in fracture morphology, influenced by both the type and concentration of the filler. Neat PLA exhibits a uniform, smooth, and transparent structure, indicative of high material homogeneity and cohesion.

Upon the incorporation of sorghum leaf fillers, the structure of the composites becomes significantly altered—darker, less homogeneous, and more irregular. As the filler content increases, the presence of fiber agglomerates, microcracks, and voids becomes more apparent. These features suggest poor interfacial adhesion between the filler phase and the matrix, likely resulting from limited chemical compatibility. The surface of the leaves may be coated with natural hydrophobic waxes, which hinder adequate wetting by the hydrophilic PLA matrix, leading to interfacial discontinuities and localized stress concentrators.

In composites containing stem-based fillers, the fracture surfaces appear more uniform, with better-dispersed fibers, particularly at lower filler concentrations. Although some defects and heterogeneities are also present in these samples, their intensity is lower compared to the leaf-filled counterparts. This may indicate slightly better wetting and more effective bonding between the stem fibers and the PLA matrix. However, at higher filler concentrations, a decline in structural integrity is also noticeable.

In summary, microscopic analysis confirms notable structural differences in the composites depending on the filler type and content. Composites reinforced with stem fibers exhibit a more favorable morphology than those with leaf fillers, which aligns with observations indicating better interfacial adhesion in the case of stem-derived reinforcements. The presence of voids, agglomerations, and interfacial discontinuities, particularly in leaf-derived composites, may adversely affect their mechanical performance.

**Figure 11:**
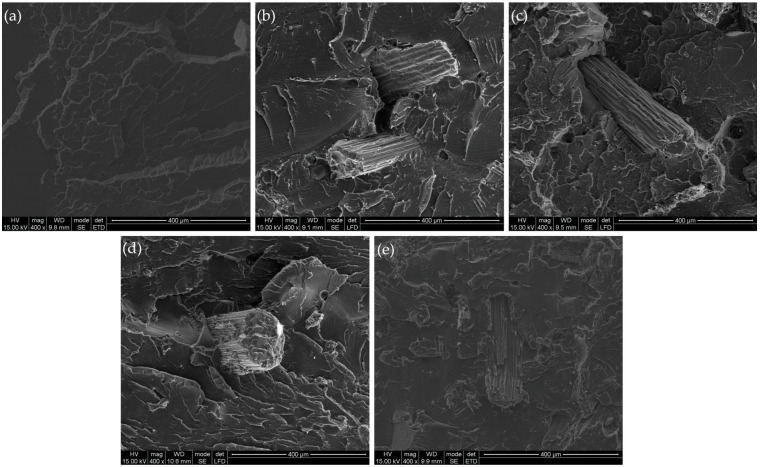
SEM images of fracture surfaces of PLA and PLA-based composites at 400× magnification: (**a**) PLA, (**b**) PLA5%L, (**c**) PLA15%L, (**d**) PLA5%S, (**e**) PLA15%S.

Fracture surface morphologies of the tested samples were further investigated using scanning electron microscopy (SEM), and representative micrographs are presented in Figure 11a–e. These high-resolution images provide additional insight into the internal structure and filler–matrix interactions.

Image (a), corresponding to neat PLA, reveals a smooth, continuous surface, typical of brittle fracture in a homogeneous and defect-free matrix. The morphology confirms high internal cohesion and uniformity of the polymer structure.

In contrast, images (b) and (c), representing composites filled with sorghum leaf fibers (L), show a heterogeneous and irregular fracture surface with visible fiber pull-outs, voids, and interfacial gaps. These features become more pronounced with increasing filler content and are indicative of poor interfacial adhesion between the PLA matrix and the leaf-derived fibers. This supports the hypothesis that surface waxes or hydrophobic layers on the leaves may reduce compatibility with the hydrophilic matrix, leading to localized stress concentrators and weaker mechanical integrity. Images (d) and (e), corresponding to composites filled with stem fibers (S), exhibit a more compact and integrated morphology. Although microvoids and some fiber pull-outs are still present, their frequency and severity are lower compared to the leaf-filled samples. The better dispersion and wettability suggest improved interfacial adhesion between the stem fibers and PLA, especially at lower filler concentrations.

These SEM results confirm the earlier microscopic observations and reinforce the conclusion that composites reinforced with stem fibers demonstrate a more favorable structural morphology, which is likely to contribute to their relatively better mechanical performance. In contrast, the presence of structural defects in leaf-filled composites could negatively influence their strength and toughness.

## 4. Conclusions

This study confirmed the potential of using sorghum-derived lignocellulosic fillers in composites from PLA filled with natural fibre. Filler type and content significantly affect material properties. Stem-derived fillers provided better stiffness–ductility balance, higher homogeneity, and improved interfacial adhesion compared to leaf-derived fillers, which tended to agglomerate and reduce tensile strength and elongation at break (possibly due to surface waxes hindering matrix wetting. All composites exhibited reduced thermal stability and impact strength with increasing filler content (varied by filler type). Overall, sorghum stems proved to be more suitable than leaves for reinforcing biodegradable PLA composites, offering more favorable performance characteristics.

From a practical perspective, sorghum is an increasingly cultivated and climate-resilient crop in Europe, and its leaves and stems represent underutilized agricultural waste. Their use in polymer composites aligns with the principles of cascaded biomass utilization and the circular economy, offering a promising path for value-added applications of non-edible plant residues beyond energy recovery.

## Figures and Tables

**Table 1:** Thermal parameters of PLA and investigated composites.

1st heating							
**Sample**	**T_g_ [°C]**	**T_M1_ [°C]**	**T_M2_ [°C]**	**T_CC_ [°C]**	**∆Hcc [J/g]**	**∆H_M_** **[J/g]**	**Xc** **[%]**
PLA	59.5	155.0	-	120.5	19.6	20.65	1.02
PLA5%L	60.8	155.5	-	111.6	25.41	25.75	0.38
PLA10%L	61.0	153.4	156.1	112.5	26.7	27.5	0.95
PLA15%L	59.6	157.9	153.0	109.2	27.01	28.5	1.88
PLA5%S	58.3	154.2	157.2	117.8	27.61	28.46	0.96
PLA10%S	59.9	152.5	157.9	111.2	27.3	29.24	2.31
PLA15%S	58.7	150.8	158.5	107.2	26.87	28.65	2.25
2nd heating							
PLA	60.7	153.9	-	125.9	11.51	11.65	0.15
PLA5%L	60.6	154.1	-	122.3	23.4	24.06	0.74
PLA10%L	60.1	150.5	155.8	113.5	28.9	29.35	0.53
PLA15%L	57.8	151.8	158.0	109.1	29.3	30.37	1.35
PLA5%S	58.7	150.3	156.3	114.6	31.31	31.42	0.12
PLA10%S	59.5	149.2	156.0	111.1	27.80	30.07	3.39
PLA15%S	58.1	148.5	155.8	113.0	28.15	30.3	2.71

**Table 2:** Thermomechanical parameters obtained from DMTA analysis.

Sample	G’ at 25 °C [MPa]	T_g_ [°C]	Tan δ
PLA	1390	68.0	2.68
PLA5%L	1060	67.0	2.39
PLA10%L	1100	66.5	2.33
PLA15%L	1090	66.3	2.25
PLA5%S	1170	66.0	2.52
PLA10%S	1380	65.0	2.35
PLA15%S	1480	65.0	2.21

**Table 3:** Mechanical properties of PLA composites with sorghum leaf (L) and stem (S) fibrous fillers—Tensile strength, Young’s Modulus, and Strain at Tensile strength (ISO 527).

Sample	Tensile Strength [MPa]	Young’s Modulus [MPa]	Elongation at Break [%]
PLA	44.00 ± 0.46	4163 ± 166	2.40 ± 0.17
PLA5%L	42.17 ± 0.45	4540 ± 105	2.13 ± 0.25
PLA10%L	35.47 ± 1.86	4997 ± 80	1.20 ± 0.10
PLA15%L	30.00 ± 1.50	5340 ± 20	0.86 ± 0.01
PLA5%S	41.63 ± 3.00	4997 ± 446	1.50 ± 0.10
PLA10%S	38.10 ± 1.18	4737 ± 271	1.57 ± 0.21
PLA15%S	37.33 ± 0.15	4440 ± 20	1.40 ± 0.10

**Table 4:** Impact strength values of PLA composites reinforced with sorghum leaf (L) and stem (S) fillers.

Sample	Impact Strength [kJ/m^2^]
PLA	10.46 ± 1.92
PLA5%L	8.16 ± 1.17
PLA10%L	7.52 ± 0.78
PLA15%L	6.98 ± 1.02
PLA5%S	5.23 ± 1.36
PLA10%S	5.03 ± 1.41
PLA15%S	4.59 ± 1.21

**Table 5:** Average density values of PLA composites reinforced with natural fillers from sorghum leaves (L) and stems (S).

Sample	Density [g/cm^3^]
PLA	1.238 ± 0.006
PLA5%L	1.221 ± 0.004
PLA10%L	1.224 ± 0.004
PLA15%L	1.235 ± 0.003
PLA5%S	1.222 ± 0.009
PLA10%S	1.251 ± 0.009
PLA15%S	1.261 ± 0.004

**Table 6:** Thermal decomposition parameters of PLA composites with sorghum leaf (L) and stem (S) fillers under nitrogen and air atmospheres.

Sample	T5% [°C]	T10% [°C]	Residual Mass [%]	DTG Peak [°C]/ Max. Rate [%/min]	T5% [°C]	T10% [°C]	Residual Mass [%]	DTG Peak [°C]/ Max. Rate [%/min]
	Nitrogen				Air			
L	147.5	205.5	24.35	314/6	166.7	220.7	5.37	293/6
S	148.0	188.0	22.99	217/3	155.2	190.7	3.72	289/5
PLA	330.7	339.8	0.20	367/32	322.5	332.9	0.25	362/31
PLA5%L	288.4	300.1	1.92	340/23	295.9	308.1	0.78	352/22
PLA10%L	285.1	295.6	2.39	333/25	289.4	301.3	0.71	345/22
PLA15%L	290.9	300.9	1.52	336/28	296.1	308.0	0.61	353/21
PLA5%S	295.3	307.8	0.57	355/21	293.5	305.8	1.39	346/24
PLA10%S	291.1	303.6	0.58	345/20	278.1	291.6	2.87	329/24
PLA15%S	269.1	284.9	1.15	320/21	270.4	283.3	3.52	321/22

## Data Availability

The original contributions presented in this study are included in the article. Further inquiries can be directed to the corresponding authors.
